# Mir-454-3p induced WTX deficiency promotes hepatocellular carcinoma progressions through regulating TGF-β signaling pathway

**DOI:** 10.7150/jca.67478

**Published:** 2022-03-21

**Authors:** Hui Liu, Juan Zheng, Shengqian Yang, Qibei Zong, Zhiwen Wang, Xinghua Liao

**Affiliations:** 1 Institute of Biology and Medicine, College of Life and Health Sciences, Wuhan University of Science and Technology, Hubei, 430081, P. R. China.; 2 Department of Joint Laboratory for Translational Medicine Research, Liaocheng People's Hospital, Shandong, 252000, P. R. China.; 3 Yueyang Key Laboratory of Chronic Noncommunicable Diseases, Yueyang Vocational & Technical College, Hunan, 414000, P. R. China.

**Keywords:** miR-454-3p, WTX, HCC, metastasis, autophagy

## Abstract

**Background:** Wilms tumor gene on X chromosome (WTX) is an X-linked tumor suppressor gene in Wilms tumor; however, however, the molecular mechanism of WTX in the occurrence and development of HCC has not been reported.

**Methods:** The expression of miR-454-3p and WTX wre analyzed in 32 matched human HCC and normal tissue samples. The molecular mechanisms of miR-454-3p/WTX/TGFβ signaling in cell proliferation, migration, invasion and autophagy were investigated *in vitro* and *in vivo*.

**Results:** WTX expression was downregulated in HCC tissues; lower WTX levels were associated with poor HCC patient outcomes. WTX loss triggers the activation of TGF-β signaling, which promotes HCC cells proliferation, migration, invasion and autophagy. Further mechanistic study showed that the aberrant upregulation of miR-454-3p was identified as the reason of WTX loss in HCC.

**Conclusions:** WTX is a tumor suppressor gene in HCC, miR-454-3p/WTX/TGFβ signaling will provide a new direction for the diagnosis and treatment of HCC.

## Introduction

Hepatocellular carcinoma (HCC) is a common malignant tumor, ranking fourth in incidence and third in mortality among tumors 1-4. Although early-stage hepatocellular carcinoma patients can be treated with surgery, 70% of patients will recurrence and metastasize within five years 5, 6. Due to the lack of therapeutic biomarkers for effective diagnosis, the overall survival rate of HCC patients is poor 7, 8. Therefore, to search for efficient therapeutic targets involving in the progression of HCC is becoming urgent.

WTX (Wilms Tumor gene on X chromosome) is an X-linked tumor suppressor discovered in the Wilms tumor 9-11. WTX forms a complex with β-TrCP2, β-catenin, APC, and AXIN1 to promote the ubiquitination of β-catenin protein, which inhibits the activation of WNT signaling pathway in Wilms tumors 12-14. Recent studies have shown that WTX can regulate the occurrence and development of gastric cancer by controlling AKT1 expression 15. However, the biological function of WTX in liver cancer is still unknown.

MicroRNAs (miRNAs) are non-coding RNA composed of 19-25 nucleotides that can complementally bind to the mRNA of target genes and inhibit post-transcriptional processes 16, 17. More and more studies show that miRNAs play important biological functions in the pathogenesis of tumors, indicating that they can be used as new targets for tumor diagnosis and treatment 18-20. Mir-454-3p has been confirmed to be abnormally expressed in a variety of cancers, down-regulated in non-small cell lung cancer and breast cancer, and up-regulated in glioma and cervical cancer 21-24. However, the biological mechanisms of miR-454-3p in HCC needs further explored.

In our study, we identified to identify the regulatory mechanism of WTX in the occurrence and development of HCC. We found that downregulation of WTX in HCC was due to the abnormal increase of miR-454-3p, which is related to the poor prognosis of patients. WTX loss promoted HCC cells proliferation, migration, invasion and autophagy through the activation of the TGF-β signaling. And we determined that miR-454-3p/WTX/TGF-β signaling axis regulates the occurrence and development of HCC.

## Methods

### Tissue specimens

In total, 32 matched human HCC and normal liver samples were collected from patients undergoing liver cancer resection at Liaocheng People's Hospital (Liaocheng, P. R. China). The collection and use of specimens were carried out with the informed consent of all patients and the approval of the Medical Ethics Committee of Liaocheng People's Hospital.

### Cell culture

HepG2 and Huh7 cell lines were purchased from American Type Culture Collection (Manassas, VA). All cell lines were cultured in DMEM (DMEM, BI, Israel) supplemented with 10% FBS at 37 °C in a 5% CO2 humidified incubator.

To generate stable WTX overexpressing cells, we infect HCC cells with a lentivirus expressing WTX or a negative control (Genechem, Shanghai, China), and use 500 ng/mL puromycin for resistance selection. After 6 days, the cells were collected and verified by real-time RT-PCR.

### Plasmids, siRNAs, miRNAs, and transfection

The human WTX overexpression plasmid pcDNA3.1-WTX was obtained from Origene (Beijing, China). siRNA-WTX (si-WTX), miR-454-3p mimic and miR-454-3p inhibitor were obtained from RiboBio (Guangzhou, China). Target sequences of siRNAs were as follows: siWTX (UAUGCCAGGGAGGCCCACA), and siCon (AATTCTCCGAACGTGTCACGT).

### qRT-PCR

RNA was extracted using RNAiso-Plus (TAKARA, DaLian, China), 1 ug RNA was synthesized into cDAN using PrimeScript RT reagent Kit (Promega), and SYBR Green SuperMix (Tiangen, China) was used for qRT-PCR. Primer sequences are as follows: WTX forward, 5' CAGCTCAGGGAGGTTTTGAG-3' and 5'-CCAGACATGCAAGAAGCAAA-3'; GAPDH forward, 5'- ATGACATCAAGAAGGTGGTG -3' and 5'- CATACCAGGAAATGAGCTTG -3'.

### Transwell invasion and migration assays

Chamber invision ability were performed with the transwell 24-well Boyden chamber (Corning, USA). 5×10^4^ cells were seeded into chambers and incubated for 24 h. The cells were fixed with formaldehyde for 20 minutes, stained with 0.1% crystal violet and photographed under a microscope for statistics.

### Cell proliferation and scratch-wound assays

Cell proliferation was measured using MTS Assay Kit (Sigma). For cell scratch-wound assays, cells were inoculated in 6-well plates and transfected 12 hours later. A horizontal line was drawn on the monolayers with a 200 µL tip. At 0, 24 and 48 h, they were photographed and analyzed under a microscope.

### EdU assay

HCC cells were inoculated in 24-well plates, transfected after 12 hours, and then transfected with 50 μM edu was incubated and finally fixed with 4% paraformaldehyde. The images were taken and analyzed under confocal laser microscope.

### Autophagy assay

The HCC cells were inoculated in 24-well plates, and 48 hours after transfection, the cells were stained with CYTO-ID autophagy detection kit. The images were taken and analyzed under confocal laser microscope.

### Western blotting

Western blot analysis was performed as described previously 25. Primary antibodies used were anti-WTX (A20434; Abclonal), anti-TGF-β2 (A3640; Abclonal), anti-phospho-SMAD3 (AP0727; Abclonal), anti-SMAD3 (A19115; Abclonal), anti-LC3B (A19665; Abclonal) and anti-GAPDH (A19056; Abclonal).

### Immunohistochemistry

Firstly, paraffin sections were dewaxed and gradient dehydrated, then antigen repaired, and finally stained.

### Animal studies

All animal experiments were conducted in accordance with the guidelines of the Animal Care Committee of Wuhan University of science and technology (P. R. China). The mice used in this experiment were all male BALB/c nude mice aged 4 weeks. All mice were purchased from Charles River Laboratories (P. R. China) and raised in a sterile animal room. HepG2 or Huh7 stable cell lines with a total amount of 1×10^7^ cells were subcutaneously injected into the lower right flank of Nude mice. All mice were euthanized 28 days later and tumors were excised carefully and final tumor weight measured.

### Statistical analysis

All experiments were independently repeated more than three times, and the experimental results were expressed as means ± SEM. Statistical analysis was performed using the SPSS20 software (SPSS, USA). P < 0.05 was defined as statistically significant.

## Results

### WTX is downregulated in HCC tissues

**Figure 1:**
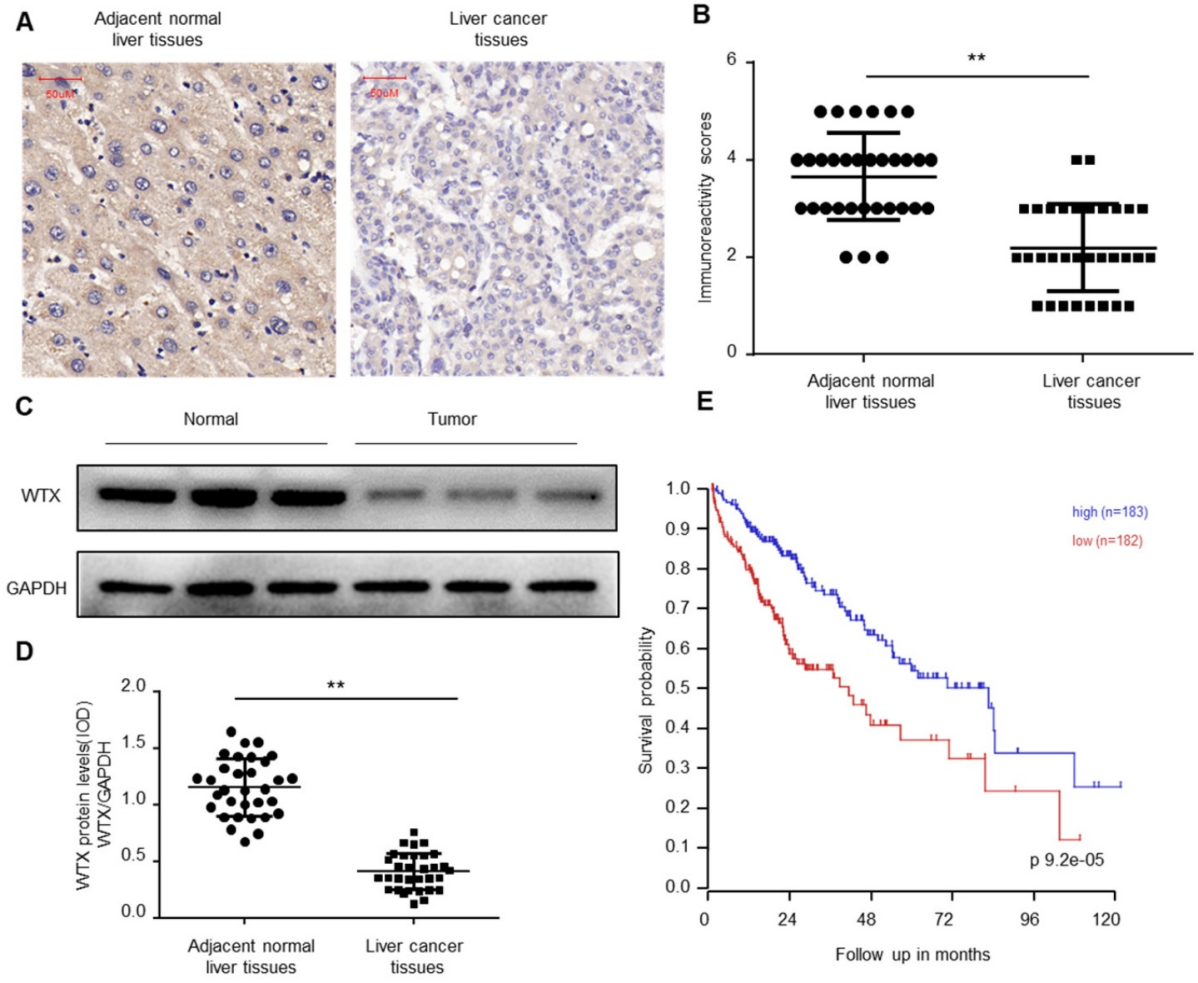
** WTX is downregulated in HCC. (A, B)** Immunohistochemistry of WTX expression in 32 HCC and paired adjacent normal liver tissue samples. Representative immunohistochemistry images (A) and semi-quantitative evaluation (B) of WTX protein expression. **(C, D)** Analysis of WTX expression in 32 HCC and paired adjacent normal liver tissue samples. Representative western blotting images of WTX protein levels in three normal liver tissues and three HCC tissues (C). WTX and GAPDH protein levels were determined via densitometry using ImageJ and are represented as IOD (D). **(E)** Kaplan-Meier survival analysis of HCC patients with WTX high or WTX low expression. Data represent the means ± SEM. **P < 0.01. ns, not significant.

To explore the biological function of WTX in Hepatocellular carcinoma (HCC), immunohistochemical (IHC) staining was performed on the collected 32 HCC and paired normal tissues. The results of immunohistochemistry showed that WTX expression was downregulated in HCC tissues (Figure 1A-B). Western blotting was further implemented and verified WTX loss in HCC (Figure 1C-D). Kaplan-Meier survival curves showed that lower WTX expression levels in HCC tissues were significantly associated with poor prognosis of HCC patients (Figure 1E). It suggests that WTX can be a new target for diagnosis and treatment of HCC.

### WTX inhibits the malignant potential of HCC cells

**Figure 2:**
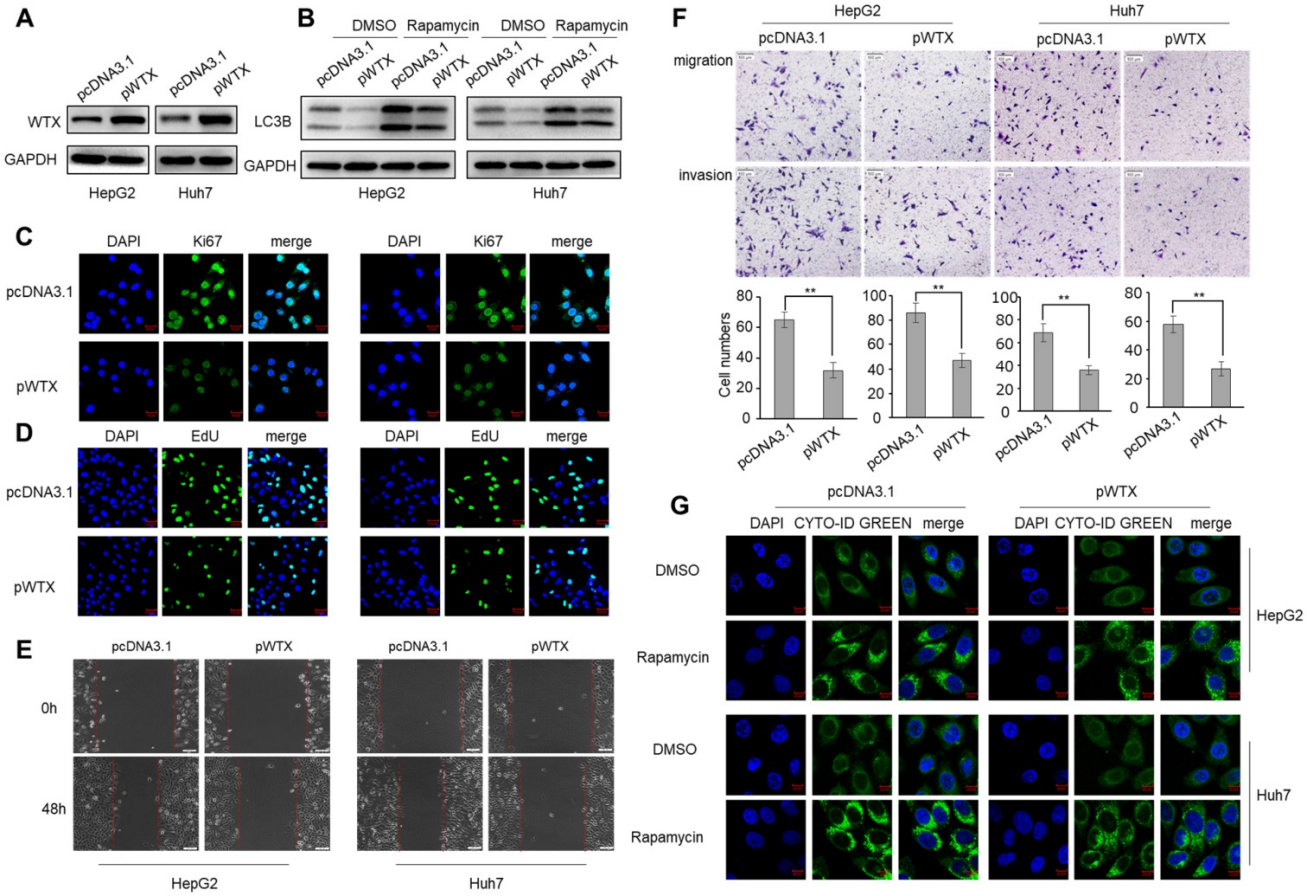
** WTX inhibits HCC cells proliferation, migration, invasion and autophagy.** HepG2 and Huh7 cells were transduced with WTX expression plasmid (pWTX) or pcDNA3.1 as indicated. **(A)** Levels of WTX were detected by western blot. **(B)** Effects of overexpression of WTX on protein levels of LC3B in HCC cells. **(C)** The expression levels of the cell proliferation marker Ki67 were detected by immunofluorescence. **(D)** Effect of WTX on cell proliferative abilitys was examined by EdU incorporation assay. **(E, F)** Cell metastasis was determined by Scratch wound assays (E) or Transwell migration and Matrigel invasion assays (F). **(G)** Cell autophagy was detected using an autophagosome detection kit. Data represent the means ± SEM. **P < 0.01.

To explore the biological mechanism of WTX in HCC, HepG2 and Huh7 cell lines were transfected with WTX expression plasmid (pWTX) or control plasmid (pcDNA3.1) (Figure 2A). MTS assay showed that WTX inhibited the proliferation of HCC cells (Figure S1A). IF staining also revealed that WTX negatively regulated Ki67 (a cell proliferation marker) protein expression in HCC cell lines (Figure 2C). Furthermore, EdU assays showed that DNA replication in WTX-overexpressing HCC cells was significantly reduced (Figure 2D). In an *in vitro* wound-healing assay, compared with control cells, the overexpression of WTX inhibited the migration ability of cells (Figure 2E). As shown in Figure 2F, cell migratory and invasive ability were significantly inhibited compared with control cells. Interestingly, we also found that WTX inhibited rapamycin-induced autophagy (Figure 2B and G). In addition, WTX knockdown promoted proliferation, migration, invasion and autophagy in HCC cells (Figure S2).

**Figure 3:**
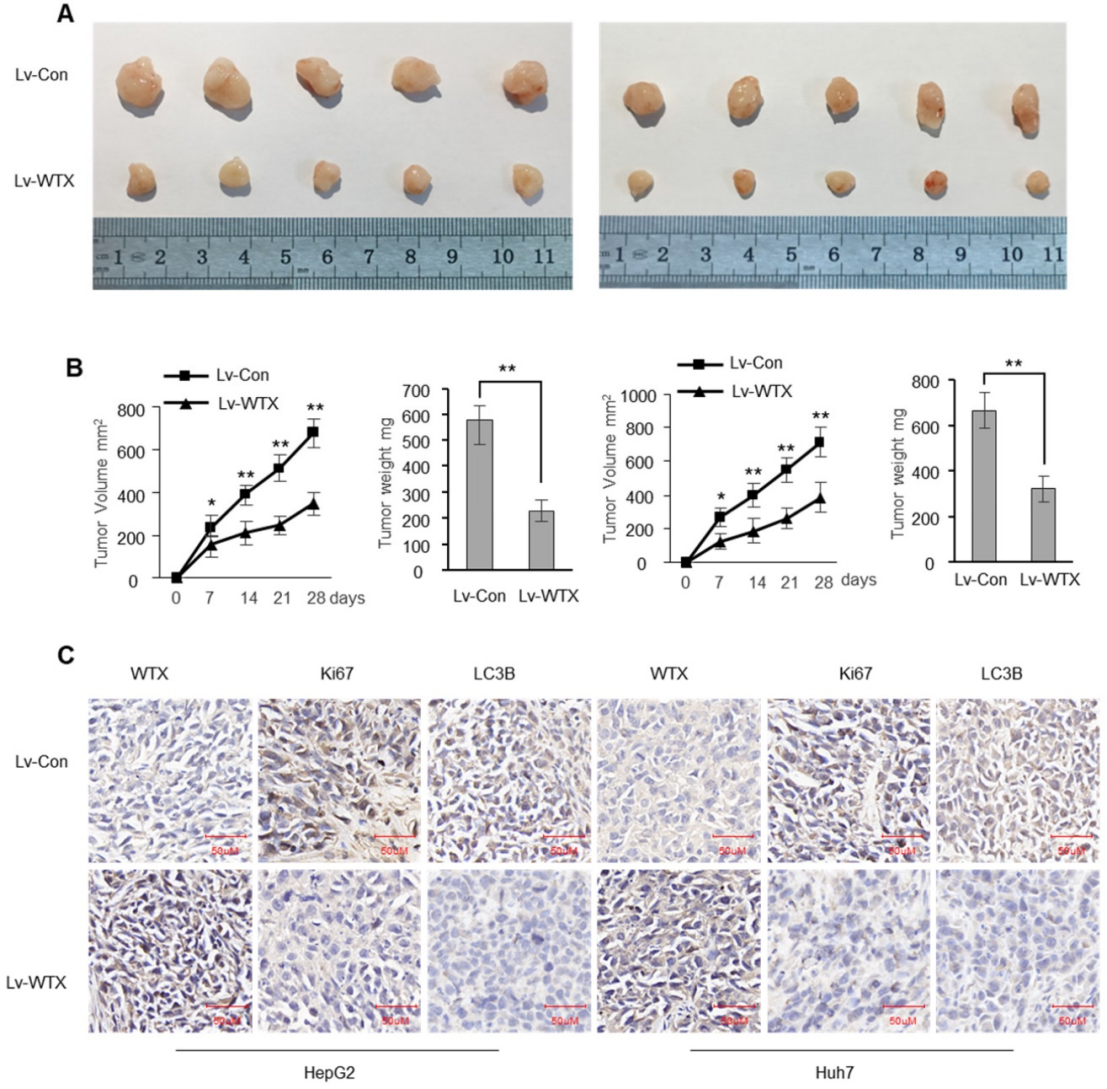
** WTX suppresses liver tumor growth *in vivo*.** Subcutaneous xenografts of HCC cells infected with WTX overexpressing lentivirus (Lv-WTX) or control lentivirus (Lv-Con). **(A)** Images of the tumors at autopsy from nude mice are presented. **(B)** Tumor volumes and average weight of xenografted tumors were measured. **(C)** Immunohistochemical (IHC) staining of WTX, Ki67 and LC3B in xenografted tumors from Lv-WTX cells or control cells. Data represent the means ± SEM. **P < 0.01.

To investigate whether WTX inhibits tumorigenicity *in vivo*, we constructed stable WTX-overexpressing cells (Lv-WTX-HepG2 and Lv-WTX-Huh7) and control cells (Lv-Con-HepG2 and Lv-Con-Huh7). We injected Lv-WTX cells or Lv-Con cells subcutaneously into the right abdomen of nude mice. All mice were euthanized 28 days later. As expected, the average volume and weight of the tumors injected with Lv-WTX cells were significantly reduced (Figure 3A-B). In addition, Ki67 and LC3B in the experimental group tumors were significantly reduced (Figure 3C).

### Aberrant miR-454-3p upregulation relates to WTX loss in HCC

**Figure 4:**
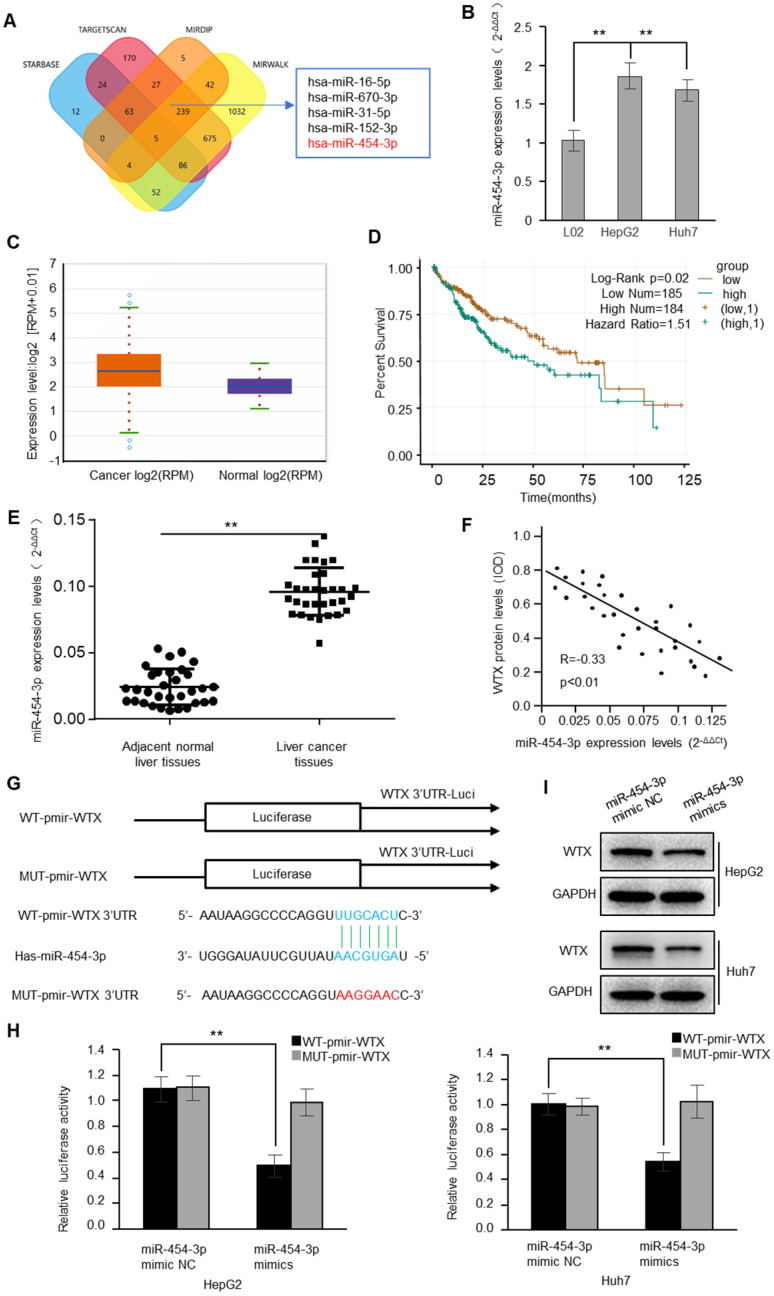
** WTX is a direct target of miR-454-3p. (A)** The four-way Venn diagram reveals the numbers of overlapping miRNAs obtained using four publicly available bioinformatics algorithms and the microarray-based WTX signature. **(B)** Real-time RT-PCR was used to detect the relative expression of miR-454-3p in normal liver cells and HCC cells. **(C)** Expression of miR-454-3p in normal tissues and HCC tissues from the Oncemine database. **(D)** Kaplan-Meier survival analysis of HCC patients with miR-454-3p high or miR-454-3p low expression. **(E)** Analysis of miR-454-3p expression in 32 HCC and paired adjacent normal liver tissue samples. **(F)** Correlation between miR-454-3p levels and WTX levels in 32 HCC tissues. **(G)** Nucleotide predicted miR-454-3p-binding site in the WTX mRNA 3′-UTR. **(H)** Luciferase activities were measured in HepG2 and Huh7 cells transfected with reporter plasmids containing WT-pmir-WTX or MUT-pmir-WTX together with miR-454-3p mimics or miR-454-3p mimic NC. **(I)** Levels of WTX were detected by western blot. Data represent the means ± SEM. **P < 0.01.

To understand the regulatory mechanism of WTX, we used bioinformatics algorithms to predict 5 potential binding miRNAs in WTX 3'-UTR (Figure 4A). Interestingly, only miRNA-454-3p was significantly increased (Figure 4B). The analysis of starBase showed that the expression of miRNA-454-3p in the tissues of HCC patients was higher than that of normal tissues (Figure 4C). The survival analysis from the TCGA database also showed that the survival of HCC patients with high expression of miR-454-3p was poor (Figure 4D). Similarly, by detecting the 32 cases of HCC and matched normal liver tissue samples, we found that miR-454-3p in HCC was significantly higher than that in neighboring tissues, which was negatively correlated with the expression of WTX (Figure 4E-F). To verify whether miR-454-3p can directly bind to the WTX3′UTR, luciferase assays were performed (Figure 4G). The results conrmed that miR-454-3p could directly regulate WTX (Figure 4H). In addition, after miR-454-3p mimics transfection, WTX was also significantly reduced in HCC cells (Figure 4I).

### miR-454-3p mimics the effect of knockdown of WTX in HCC cells

**Figure 5:**
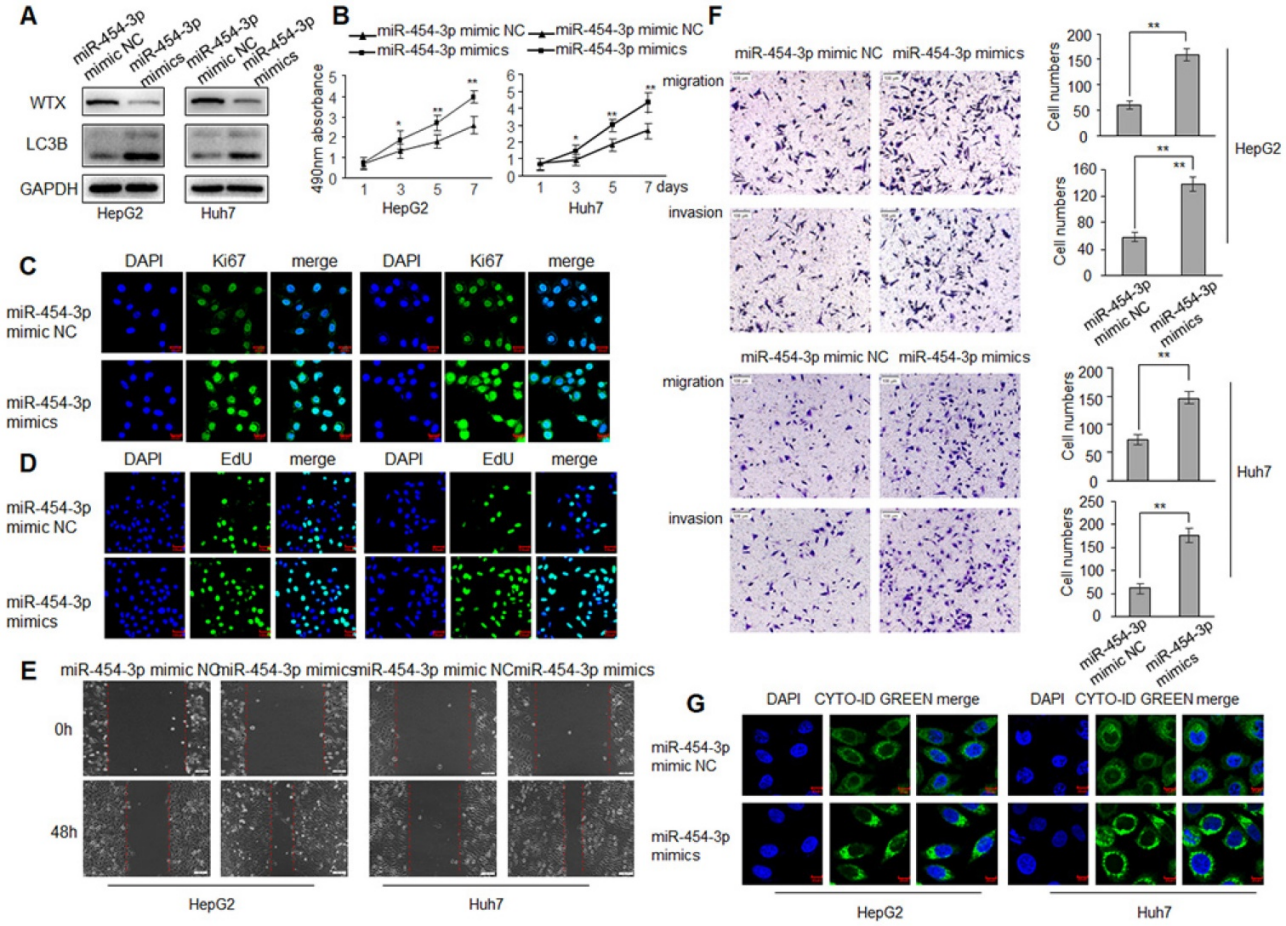
** miR-454-3p promotes the proliferation, migration, invasion and autophagy of HCC cells.** HepG2 and Huh7 cells were transduced with miR-454-3p mimic NC or miR-454-3p mimics. **(A)** Levels of WTX and LC3B were detected by western blot. **(B)** MTS assay indicted that miR-454-3p mimics promotes ability of proliferation. **(C)** The expression levels of Ki67 were detected by immunofluorescence. **(D)** Effect of miR-454-3p on cell proliferative abilitys was examined by EdU incorporation assay. **(E)** Cell would healling ability was improved in miR-454-3p mimics cells. **(F)** Chamber invision ability was improved in miR-454-3p mimics cells. **(G)** Cell autophagy was detected using an autophagosome detection kit. Data represent the means ± SEM. **P < 0.01.

WTX inhibits HCC progression and miR-454-3p could directly regulate WTX, we investigated whether miR-454-3p have a similar effect on HCC tumorigenesis? To test this hypothesis, HepG2 and Huh7 cells were transduced with miR-454-3p mimics or miR-454-3p mimic NC, WTX protein level was detected by western blot (Figure 5A). As expected, miR-454-3p significantly promoted the proliferation ability of HCC cells (Figure 5B-D). As shown in Figure 5E, 5F, cell migratory and invasive ability were significantly enhanced in the miR-454-3p mimics groups. Interestingly, autophagy also increased significantly in the miR-454-3p mimics groups (Figure 5G), the opposite experimental results appeared in the miR-454-3p inhibitor groups (Figure S3 and Figure S4).

### miR-454-3p -WTX axis regulates TGF-β signaling in HCC cells

**Figure 6:**
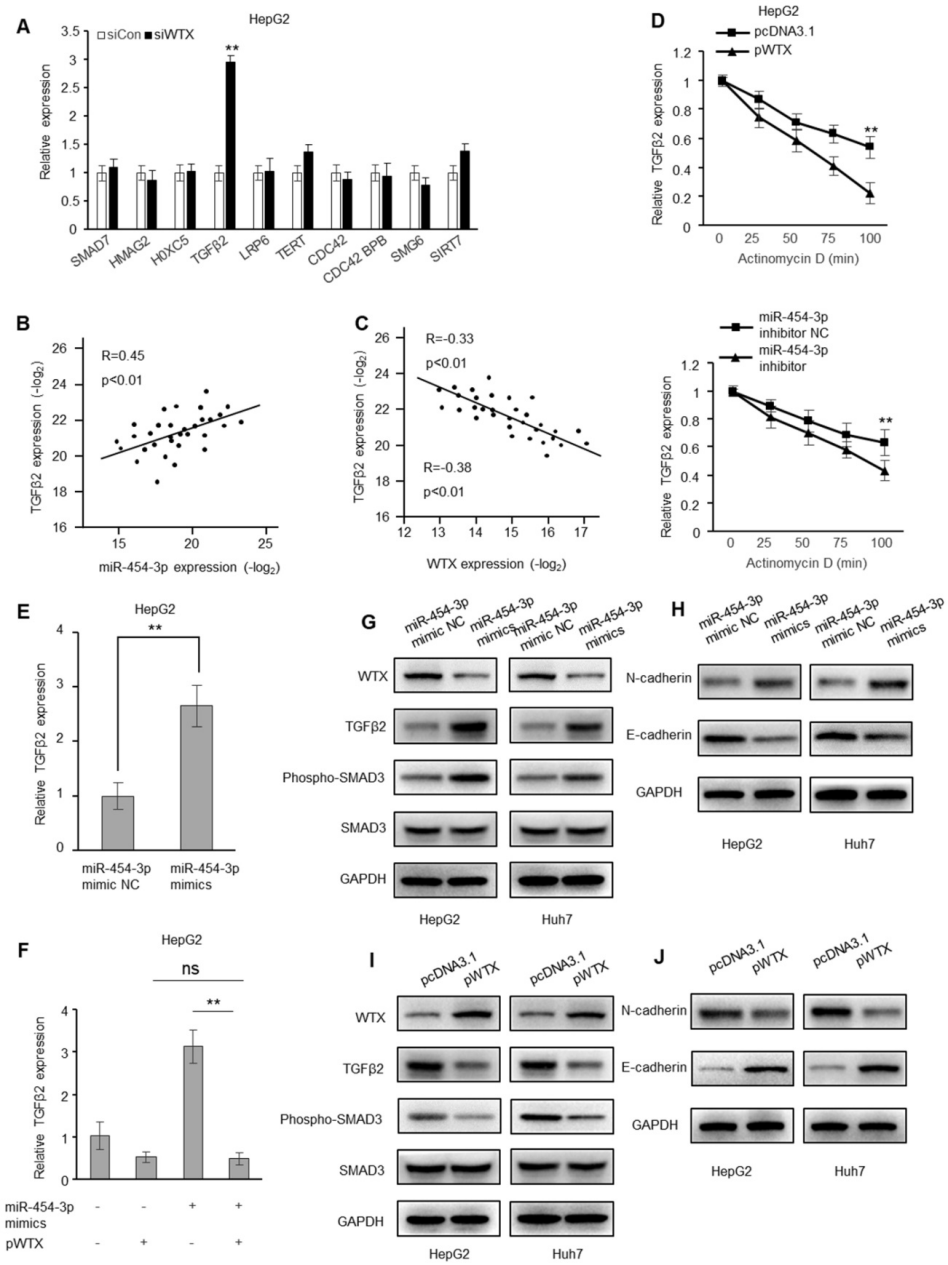
** WTX inhibits the expression of TGFβ2. (A)** RT-qPCR of selected genes related to the ranked pathways andtumorigenesis in WTX-knockdown HepG2 cells. **(B, C)** Correlation of miR-454-3p and TGFβ2 (B) and of WTX and TGFβ2 (C) expression in 32 HCC tissues. **(D)** The decay of TGFβ2 mRNA was monitored by RT-qPCR in WTX overexpression or miR-454-3p suppression HepG2 cells by blocking mRNA synthesis using actinomycin D (ActD, 5 µg/mL) for the indicated time points upon normalization to RNA input levels. **(E)** The mRNA levels of TGFβ2 were determined in HepG2 cells transfected with miR-454-3p mimics. **(F)** The mRNA levels of TGFβ2 were determined in HepG2 cells cotransfected with pWTX and miR-454-3p mimics. **(G)** The TGFβ2 protein and phosphorylated SMAD3 levels were determined in miR-454-3p-overexpressing HCC cells. **(H)** The N-cadherin and E-cadherin levels were determined in miR-454-3p-overexpressing HCC cells. **(I)** The TGFβ2 protein and phosphorylated SMAD3 levels were determined in WTX-overexpressing HCC cells. **(J)** The N-cadherin and E-cadherin levels were determined in WTX-overexpressing HCC cells. Data represent the means ± SEM. **P < 0.01.

Genes related to tumorigenesis were verified by RT-qPCR. Obviously, only TGF-β2 is regulated by WTX (Figure 6A and Figure S5A). In addition, a positive correlation between TGFβ2 and miR-454-3p was observed in HCC tissues, and a negative correlation with WTX (Figure 6B-C). In addition, after WTX overexpression or miR-454-3p inhibition, TGFβ2 mRNA decays faster, indicating that they both interfere with the conversion of TGFβ2 mRNA (Figure 6D and Figure S5B). miR-454-3p mimics caused an increase in TGFβ2 mRNA levels (Figure 6E and Figure S5C), which was restored by overexpression of WTX (Figure 6F and Figure S5D). In addition, after transfection of miR-454-3p mimics, the levels of TGFβ2, N-cadherin and phosphorylated SMAD3 increased, and E-cadherin decreased (Figure 6G-H). In overexpressing WTX cells, the levels of TGFβ2, N-cadherin and phosphorylated SMAD3 decreased, and E-cadherin increased (Figure 6I-J). Collectively, miR-454-3p activates TGFβ2 signaling by targeting WTX in HCC cells.

## Discussion

In this study, we demonstrated that high expression of WTX inhibited the malignant potential of HCC cells. In addition, through the analysis of clinical samples, we found that WTX expression is reduced in HCC tissues, and lower levels of WTX are associated with poorer prognosis of HCC patients. Our data shows that WTX acts as a tumor suppressor gene in HCC, which is consistent with previous reports on the biological functions of WTX in several other cancers, including gastric cancer 15, colon cancer 11, and cervical cancer 26. These results indicate that WTX can be used as a new target for tumor prevention, diagnosis and treatment.

This study found the biological function of WTX in HCC for the first time. Our data showed that decreased WTX expression could activate TGF-β2 signal transduction in HCC cells. It is well known that abnormal activation of TGF-β2 signal can lead to tumor development and metastasis 27-30. Therefore, regulating the activity of TGF-β2 signaling may be one of the biological functions of WTX in HCC. RT-qPCR and Western blot farther conrmed that WTX negatively regulated TGF-β2 signaling activity and inhibited HCC proliferation. Our research showed that the absence of WTX could cause abnormal activation of TGF-β2 signal in HCC, thereby promoting the occurrence and development of HCC.

Abnormal expression of miRNA often leads to the silencing of target genes 17, 31, 32. To explore the molecular mechanism leading to the loss of WTX, we screened miR-454-3p as a candidate for regulating WTX expression through bioinformatics algorithms. It was reported that miR-454-3p is up-regulated in breast cancer 22, nasopharyngeal carcinoma 33 and cervical cancer 24, and plays a role as an oncogene. We haved verified that miR-454-3p could directly regulate WTX through luciferase activity experiments. Our research confirms that the miR-454-3p/WTX/TGF-β2 signaling axis regulates the occurrence and development of HCC, providing new strategies and targets for the diagnosis and treatment of HCC.

In summary, our research revealed the biological function of WTX in HCC and determined the molecular mechanism of WTX loss. Mir-454-3p inhibits WTX expression, activates the downstream TGF-β2 signaling pathway, increases cell inhibits HCC proliferation, migration, invasion and autophagy. The study also showed that miR-454-3p/WTX/TGF-β2 signaling may provide a new target for the diagnosis and treatment of HCC.

## Supplementary Material

Supplementary figures.

## Abbreviations

IHC: immunohistochemistry
WTX: Wilms tumor gene on the X chromosome
IOD: integrated optical density
UTR: untranslated region
TGF-β: transforming growth factor-β
HCC: Hepatocellular carcinoma
