# Supplementary material for: Mir-454-3p induced WTX deficiency promotes hepatocellular carcinoma progressions through regulating TGF-β signaling pathway
Source: J Cancer. 2022 Mar 21;13(6):1820–9. doi: 10.7150/jca.67478 (PMC8990407; doi:10.7150/jca.67478)
Supplement: Supplementary file 1 — Supplementary figures. [file jcav13p1820s1.pdf]

Supplementary Figure 1. WTX reduces proliferation of HCC cells.

HepG2 and Huh7 cells were transduced with WTX expression plasmid (pWTX) or pcDNA3.1 as indicated. (A) Cell proliferation was determined by MTS assay. Data represent the means ± SEM. $^{**}P < 0.01$.

Supplementary Figure 2. Silencing WTX promotes proliferation, migration, invasion and autophagy of HCC cells.

HepG2 and Huh7 cells were transfected with siWTX or siCon. (A) Levels of WTX and LC3B were detected by western blot. (B) Cell proliferation was determined by MTS assay. (C) The expression levels of the cell proliferation marker Ki67 were detected by immunofluorescence. (D) Effect of WTX on cell proliferative abilitys was examined by EdU incorporation assay. (E, F) Cell metastasis was determined by Scratch wound assays (E) or Transwell migration and Matrigel invasion assays (F). (G) Cell autophagy was detected using an autophagosome detection kit. Data represent the means ± SEM. $^{**}P < 0.01$.

Supplementary Figure 3. miR-454-3p inhibitor reduces proliferation of HCC cells.

HepG2 and Huh7 cells were transduced with miR-454-3p inhibitor NC or miR-454-3p inhibitor. (A) Cell proliferation was determined by MTS assay. Data represent the means ± SEM. $^{**}P < 0.01$.

Supplementary Figure 4. miR-454-3p inhibitor reduces proliferation, migration, invasion and autophagy of HCC cells.

HepG2 and Huh7 cells were transduced with miR-454-3p inhibitor NC or miR-454-3p inhibitor. (A) Levels of WTX were detected by western blot. (B) Levels of LC3B were detected by western blot. (C) The expression levels of the cell proliferation marker Ki67 were detected by immunofluorescence. (D) Effect of miR-454-3p inhibitor on cell proliferative abilitys was examined by EdU incorporation assay. (E, F) Cell metastasis was determined by Scratch wound assays (E) or Transwell migration and Matrigel invasion assays (F). (G) Cell autophagy was detected using an autophagosome detection kit. Data represent the means ± SEM. **P < 0.01.

Supplementary Figure 5. WTX inhibits the expression of TGFβ2.

(A) RT-qPCR of selected genes related to the ranked pathways andtumorigenesis in WTX-knockdown Huh7 cells. (B) The decay of TGFβ2 mRNA was monitored by RT-qPCR in WTX overexpression or miR-454-3p suppression Huh7 cells by blocking mRNA synthesis using actinomycin D (ActD, 5 μg/mL) for the indicated time points upon normalization to RNA input levels. (C) The mRNA levels of TGFβ2 were determined in Huh7 cells transfected with miR-454-3p mimics. (D) The mRNA levels of TGFβ2 were determined in Huh7 cells cotransfected with pWTX and miR-454-3p mimics. Data represent the means ± SEM. **P < 0.01.

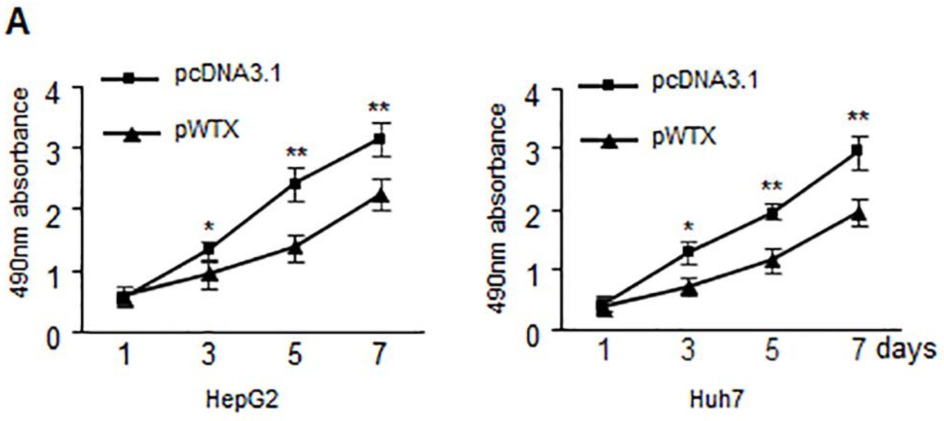

A
pcDNA3.1
pWTX
490nm absorbance
0
1
2
3
4
*
**
**
1
3
5
7
HepG2
pcDNA3.1
pWTX
490nm absorbance
0
1
2
3
4
*
**
**
1
3
5
7 days
Huh7

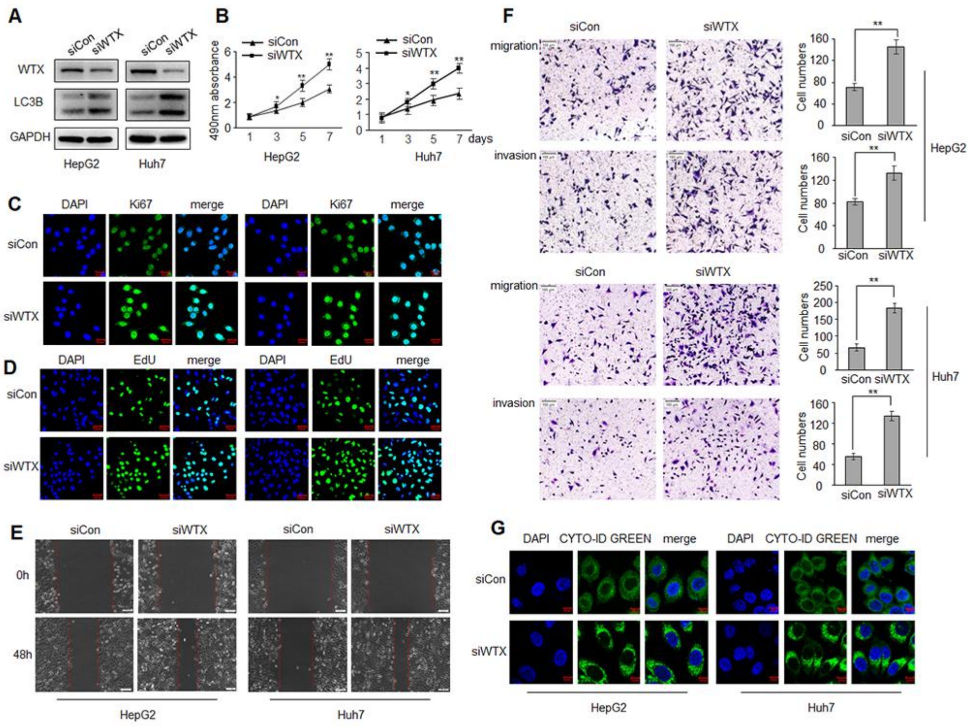
A
siCon
siWTX
WTX
LC3B
GAPDH
HepG2
Huh7
B
490nm absorbance
days
HepG2
Huh7
C
DAPI
Ki67
merge
D
EdU
E
0h
48h
F
migration
invasion
Cell numbers
G
CYTO-ID GREEN

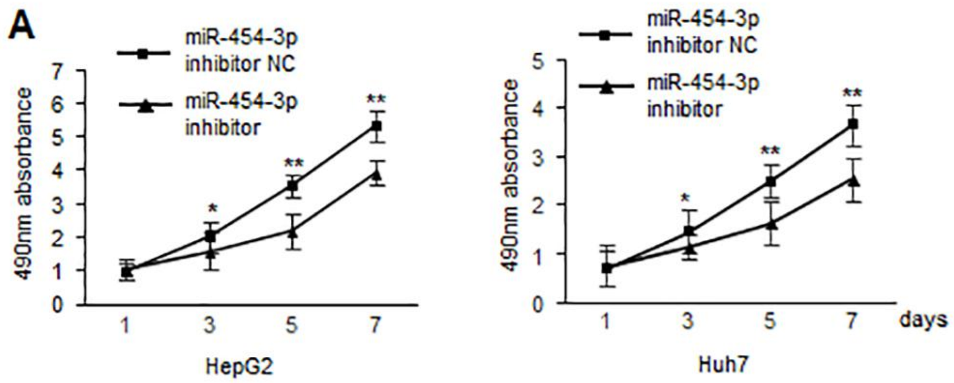
A
miR-454-3p inhibitor NC
miR-454-3p inhibitor
490nm absorbance
HepG2
Huh7
days

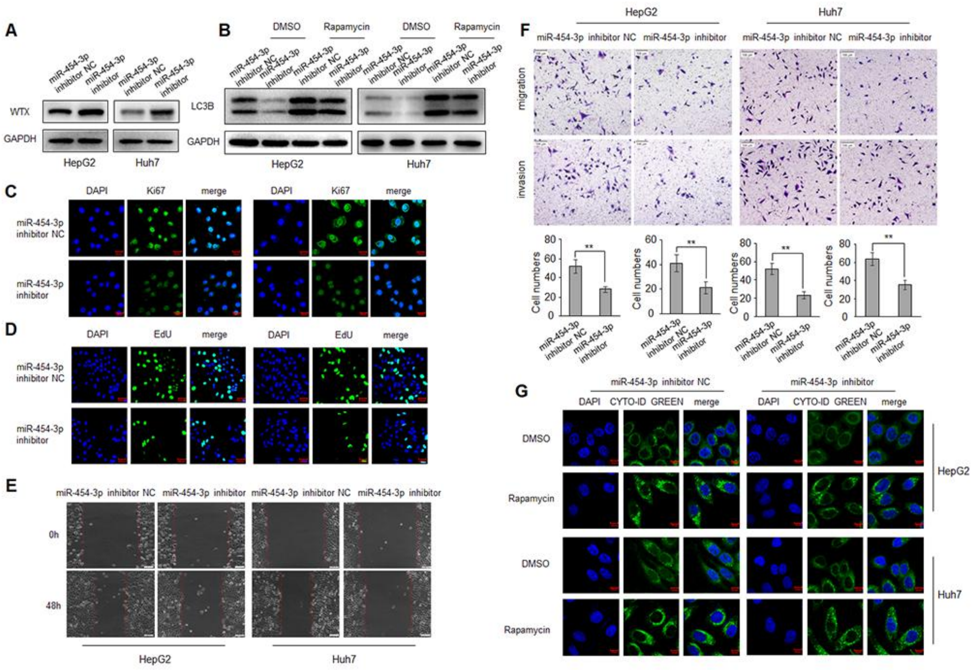
A
miR-454-3p inhibitor NC
miR-454-3p inhibitor
WTX
GAPDH
HepG2
Huh7
B
DMSO
Rapamycin
LC3B
GAPDH
HepG2
Huh7
C
DAPI
Ki67
merge
miR-454-3p inhibitor NC
miR-454-3p inhibitor
D
DAPI
EdU
merge
miR-454-3p inhibitor NC
miR-454-3p inhibitor
E
miR-454-3p inhibitor NC
miR-454-3p inhibitor
0h
48h
HepG2
Huh7
F
HepG2
Huh7
miR-454-3p inhibitor NC
miR-454-3p inhibitor
migration
invasion
Cell numbers
**
G
miR-454-3p inhibitor NC
miR-454-3p inhibitor
DAPI
CYTO-ID GREEN
merge
DMSO
Rapamycin
HepG2
Huh7

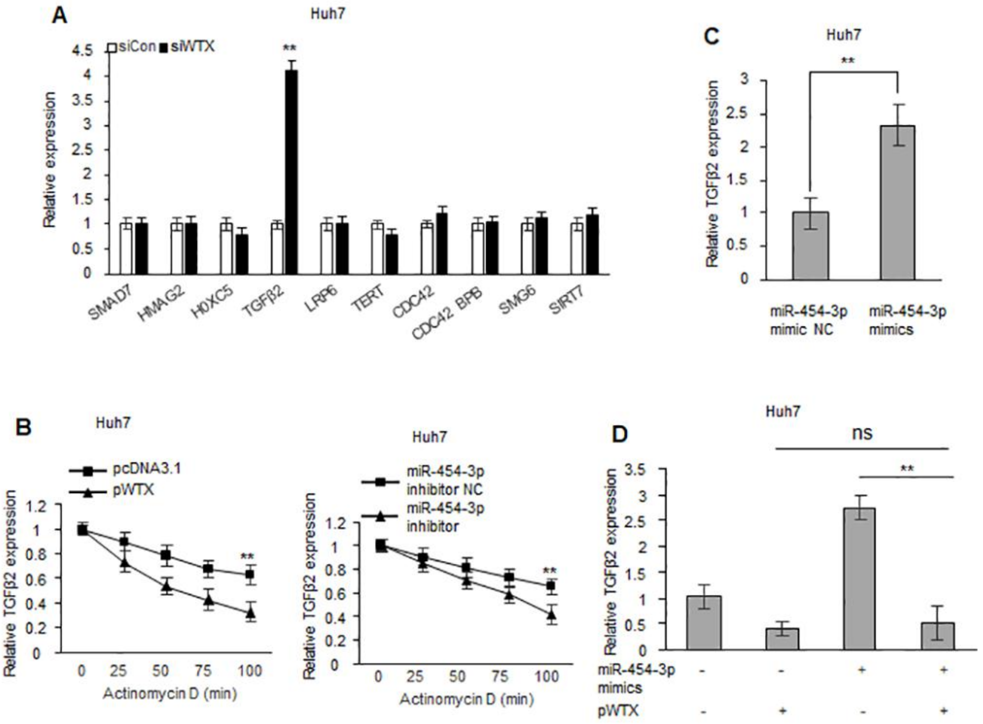
A
Huh7
siCon
siWTX
Relative expression
**
SMAD7
HMAG2
H0XC5
TGFβ2
LRP6
TERT
CDC42
CDC42 BPB
SMG6
SIRT7
B
Huh7
pcDNA3.1
pWTX
Relative TGFβ2 expression
**
Actinomycin D (min)
Huh7
miR-454-3p inhibitor NC
miR-454-3p inhibitor
Relative TGFβ2 expression
**
Actinomycin D (min)
C
Huh7
**
Relative TGFβ2 expression
miR-454-3p mimic NC
miR-454-3p mimics
D
Huh7
ns
**
Relative TGFβ2 expression
miR-454-3p mimics
pWTX
